# A Clinical Lecture on the Disinfection and Care of the Hands

**Published:** 1906-06-30

**Authors:** H. G. Kyle

**Affiliations:** Surgeon, Bristol General Hospital.


### Hospital Clinics.

### A CLINICAL LECTURE ON THE DISINFECTION AND CARE OF THE HANDS.

By H. G. Kyle, Surgeon, Bristol General Hospital.

The subject will be dealt with under four headings, namely:—

1. The sources of infection.
2. The locality of germs on the skin.
3. The disinfection of the skin.
4. Prophylaxis, or the avoidance of infection.

### 1. The Sources of Infection.

These are universal, the hand in everyday use is always septic, but it is specially in hospital that our hands receive the worst infection. The constant presence of septic wounds, and the constant dissemination of septic material from such wounds makes the hand of the hospital worker a very dangerous member. It is not only by actual contact with wounds, but also indirectly, that our hands are continually being infected.

### 2. The Locality of the Germs on the Skin.

This subject deserves careful study, so that our efforts to dislodge the infective organisms may be intelligently directed. Four principal localities require attention: (*a*) The general surface of skin; (*b*) the region of the nails; (*c*) small wounds and fissures; (*d*) the sebaceous follicles and sweat-ducts.

(*a*) *The General Surface of the Skin.*—The great majority of organisms are found here, lying upon the surface of the epidermic scales or lodged in the spaces beneath partially detached scales, the organisms are mixed up with the fatty material secreted by the sebaceous glands and thus more firmly retained in position. The tips of the fingers and the flexor aspects are the parts richest in germs. If the skin is smooth and in good condition they do not penetrate very deeply, but if the hand is rough the penetration is naturally deeper and the foreign material more difficult to remove.

(*b*) *The Region of the Nails.*—If we were asked to name the part of the hand most difficult to clean, we should without hesitation reply, "the space beneath the nails." But judging from methods frequently adopted to clean this space, I would say that many do not really understand wherein the difficulty lies. Now it is not the under-surface of the nail, nor yet the smooth skin covered by the free end of the nail that presents the difficulty. If the nails are cut moderately short the space beneath can be easily reached by the scrubbing brush. The nail itself is hard and smooth, and therefore a structure more easily cleansed than skin. Where then lies the difficulty? If we examine the space beneath the nail with the aid of a lens, or better still, a longitudinal microscopic section of the nail and its substratum, we observe that at the junction between the nail-bed and the skin of the finger the corneal layer is very rough and forms deep irregular folds; this is the part that presents the obstacle in the way of cleaning, and it is important for us to know this, because ignorance on this point is responsible for two errors that are sometimes made in cleaning the nail. The first is the use of knives, scissors, and sharp implements for the purpose; no doubt they are very effective in removing any visible dirt, but quite ineffective in penetrating the minute and irregular folds in the area under consideration, and they are worse than ineffective, for they make this area rougher still, and so capable of taking up and retaining more infection, and if the under-surface of the nail is scraped, instead of being smooth and easily cleansed, it is made rough and impossible to clean. The second error which is sometimes, though rarely made, is to cut the nails so short that the junction of nail-bed and skin is exposed; we shall see that this cannot readily facilitate the cleansing, but worse than that, it exposes this dangerous area to the wound. Normally the over-lapping nail forms a protection and prevents actual contact of this part with the wound, and probably what infective material is not removed by scrubbing will not be very largely removed in the course of an ordinary operation, certainly less will be removed under the protection of the nail than if this area were actually exposed.

The free margins of the nails are usually somewhat chipped and fissured and dirt is consequently lodged in the cracks, this can be removed by trimming the nails before operation. At the base of the nails and at the sides the skin is apt to be loose and torn and thus very difficult to clean.

(*c*) *Small Fissures and Wounds of the Epidermis.*—It is impossible to disinfect with certainty any actual visible wound of the skin, and the existence of such a wound necessitates some impervious covering, either rubber or collodion. If the wound is inflamed the hand is not fit for operation, and even with india-rubber gloves there is a risk from contamination of the glove in putting it on, or from accidental puncture or tear. But small invisible fissures are frequently present; into these foreign matter penetrates deeply and cannot be dislodged by any cleansing process; but during the squeezing and manipulation in the course of an operation this infective material is very apt to be set free.

(*d*) *Orifices of the Sebaceous Follicles and Sweat-ducts.*—Germs are relatively seldom found in either of these situations, they certainly do not appear to penetrate to any depth, for the reason that the orifices of the former are filled with sebaceous material and the contents of both are continually being forced out, which militates against the entrance of foreign material. Organisms have, however, been demonstrated in these situations occasionally, so that they are a source of danger, although not to be compared with the small fissures and artificial openings referred to above.

### 3. The Method of Disinfection.

Having studied the conditions under which organisms exist on the hands, we are now in a position to consider the means to be adopted for their removal, and firstly let me impress upon you the uselessness of dipping the hands into antiseptic solutions. When you consider the environment of the germs on the skin, hidden under epithelial scales and embedded in fatty material, it must be very evident to you that a watery solution of an antiseptic cannot reach them. But this unintelligent use of antiseptics is so wide spread and so slow to die out that I think it is necessary to call your attention to it. For a germicidal solution to be of any use, the skin must be prepared so that the solution can soak in and reach the germs upon which it is intended to act. We rely more in the present day upon the results of mechanical cleansing than upon the use of chemical germicides. Our aim is to remove the germs from the skin and to endeavour to act upon the remainder that cannot be removed by the use of antiseptics.

Now, to remove the germs we must remove the superficial epithelial scales and get rid of all fatty material; when we have done this we have removed the bulk of infective organisms. The principal agents employed for this purpose are hot water, soap, and a brush. The hot water softens both the superficial epidermis and the fatty matter, facilitating their removal; the soap assists this action and partly saponifies the fats; the brush by its mechanical action completes the removal, and the debris must be frequently washed away by several changes of water or by washing under running water. If running water is used the taps are generally regulated by a pedal, or a handle turned by the elbow, so that the fingers are not contaminated by touching them. There is no virtue in running off gallons of water during the time of washing, it is very wasteful of water, and the hands should not be most of the time under the stream, for a good lather of soap is most important in order to obtain the maximum cleansing effect, this cannot be obtained if the hands are continually under the tap, so turn off the tap when it is not required.

Have a regular routine for going over the hand and forearm, so that no part is neglected; take each finger in order, scrubbing all sides of it and under and around the nails.

Before using the sterile brush give your hands a preliminary wash to remove as much gross dirt as possible; there is always a risk of reinfecting the hands towards the end of the washing process by infective material imparted to the brush in the earlier stages. The scrubbing process should occupy at least eight minutes.

After this, a good rubbing in a sterile towel is useful to further remove any loosened epidermic scales. After the mechanical cleansing we endeavour to destroy any germs that remain by the use of some chemical disinfectant; for this to act effectively we want to ensure as far as possible that it shall penetrate and come into contact with any septic material; for this purpose we treat the hands with alcohol. Alcohol has a fourfold action:—

1. It removes any traces of grease left after the washing process.

2. It has a strong dehydrating power causing the epidermis to take up much more readily an aqueous solution of an antiseptic.

3. It has a germicidal action.

4. It hardens the epidermis and fixes the cells, thus diminishing the liability to their becoming detached during the operation.

It acts more efficiently when somewhat dilute, the hands should be wet, therefore, before using it. The treatment with alcohol should occupy two or three minutes. It is not only unnecessary, but wasteful, to have a bowlful of spirit poured out and then thrown away, for you do not want to dip your hands in, but to scrub them with the spirit. A small quantity is sufficient, and in this should be a sterile brush or piece of gauze, with which the hand and forearms should be scrubbed. The antiseptic solution is next used, this should be applied by rubbing in with gauze or a brush for three minutes. A solution of biniodide of mercury 1—1,000 is probably the best, it has a great advantage over the perchloride in that it does not coagulate albumen, and therefore has a greater penetrating power, and it does not damage the skin so much. In selecting an antiseptic each individual must consider his own idiosyncrasy; any chemical which injures the skin and leaves it rough and impossible to cleanse subsequently is worse than useless. Many surgeons advocate a solution of biniodide in spirit 1—500, so that the treatment with alcohol and antiseptic is carried out by the one procedure.

This completes the preparation of the hands, let us here, then, examine into the results obtained by our cleansing process. Have we rendered our hands sterile? Unfortunately this question must be answered in the negative. The best authorities are now agreed that it is impossible to render the hands sterile. It may be impossible to obtain any organisms from the freshly cleansed hand, but if the same hand be examined at the end of an operation, numerous cultures may be obtained, and experiments show that this reinfection comes from the hands themselves and not from the wound or the air. One of the principal factors at work in producing this reinfection is the macerating effect of blood and albuminous fluids; this, in conjunction with the rubbing and squeezing to which the hands are subjected during the operation, further detaches epidermic scales and exposes organisms which the cleansing process failed to remove. Moreover, the activity of the sebaceous and sweat glands during the operation may liberate some hidden germs. But you have often heard, I am sure, investigators claim to render their hands sterile by some particular process; I think I am right in saying that if the experiments carried out by such investigators be examined, some flaw can be discovered in every one. It is impossible in the time at my disposal to enter into the details of bacteriological investigations, but let me tell you some of the principal errors that have been made: Firstly, the antiseptic has not been properly removed before taking cultures, it is a difficult thing to do properly, and if any antiseptic is introduced into the culture medium it will probably inhibit growth. Secondly, the fact that cultures taken from the hand before operation are generally sterile has been used to prove that the hand is sterile, the reinfection which occurs during the operation not being taken into account.

Thirdly, the modes of taking material for culture have often been defective. If you wish to pursue this interesting and important question further you cannot do better than read Professor Hægler's "Cleansing, Disinfection, and Protection of the Hands," a translation of which, by Mr. Heron Watson, has just been published; or read Mr. Leedham Green's book on the same subject.

We have to face the fact that we cannot with any certainty render our hands sterile. Fortunately, the practical results are not as bad as might be expected from this conclusion. Healthy tissues have considerable power of destroying organisms, especially if the vitality of such organisms is lowered by treatment with antiseptics. Small doses of infection are more easily dealt with by the tissues than larger ones, and by careful cleansing we remove the bulk of the germs and lower the vitality of such as remain. But wound infection does occur, and when it does I believe it is attributable to the hands of the operator or assistants, and rarely to ligature material, which presents little difficulty in the way of sterilisation; of course, ligatures are apt to be infected by contact with the hand.

The lesson to be learnt from this is that the bare hand is a dangerous thing in a wound and should be introduced as little as possible. If you operate without gloves, the fingers should not be used for dissection, or retracting, or any manipulation which can be done by sterile instruments.

But I think we are forced to the conclusion that india-rubber gloves should be used, for with them we can insure almost absolute safety, which we certainly cannot without.

Two objections are raised against gloves, but I think they have little weight. They are these: (1) impairment of touch; (2) danger of puncture and escape of septic material from within the glove. As regards the first, the sense of touch soon becomes educated by practice and what little impairment may be found by some is, I think, quite counterbalanced by the increased security from infection. The second objection is quite unfounded; it has been proved that the septicity of the hand in a rubber glove at the end of an operation is much less than that of the bare hand—the absence of friction and absence of the macerating effect of albuminous fluids accounts for this. With practice, moreover, accidental puncture is rare, except in operations on bone, when it is certainly liable to occur.

4. Prophylaxis.

Thus, disinfection of the hand is not such an easy matter as is often supposed, and the lesson for us to learn from this is, that since we cannot satisfactorily disinfect the hand, it must be our constant endeavour to avoid infecting it. This, no doubt, is not a very easy thing to do, but a great deal may be done by cultivating careful habits; let me illustrate this by a few instances. Some men have acquired the habit of touching and handling everything that comes in their way, going up and down stairs they rub their hand along the bannister-rail, so picking up infective material and rubbing it into the skin; their hands are continually resting on tables and chairs in the out-patient rooms; in the examination of patients they touch septic sores or the neighbouring skin, when there is no necessity to do so.

Make it a rule never to touch septic wounds or dressings, such contact can nearly always be prevented by the use of instruments; if it is necessary to touch such things or if you are performing septic operations, rubber gloves should always be worn.

Should the hands come into contact with septic material wash them immediately, before the infective matter has dried and become rubbed in, it is much easier to remove while fresh. When, as dressers and house surgeons, you have a number of dressings to do, use rubber gloves, for the repeated washing and disinfection between each case will make your hands so rough that it will be impossible to render them even approximately clean. Put on the gloves dry, to avoid the macerating effect of moisture on the hands which will make them rough. You can wash and disinfect your gloved hands as often as necessary without hurting your skin. You must pay continual attention to the condition of the skin; if it is rough and chapped no cleansing process is of much use, your hands will be always septic. A useful measure is to rub lanoline well into the skin after operation, and then remove the excess with a towel. The continual removal of fatty material from the skin is bound to act injuriously upon it. A good plan is to grease the hands and wear kid gloves at night if the skin has got into bad condition.

This subject is one that requires continual thought and care. A very great responsibility rests upon us all, for, to quote the words of Professor Senn, "Dirty hands have destroyed more lives than all the implements of warfare."

